# Correlation Between Cerebral Venous Oxygen Level and Cognitive Status in Patients With Alzheimer’s Disease Using Quantitative Susceptibility Mapping

**DOI:** 10.3389/fnins.2020.570848

**Published:** 2021-01-18

**Authors:** YangYingQiu Liu, JunYi Dong, QingWei Song, Nan Zhang, WeiWei Wang, BingBing Gao, ShiYun Tian, ChunBo Dong, ZhanHua Liang, LiZhi Xie, YanWei Miao

**Affiliations:** ^1^Department of Radiology, First Affiliated Hospital of Dalian Medical University, Dalian, China; ^2^Department of Neurology, First Affiliated Hospital of Dalian Medical University, Dalian, China; ^3^GE Healthcare, MR Research China, Beijing, China

**Keywords:** Alzheimer’s disease, susceptibility weighted imaging, magnetic susceptibility value, cerebral vein, magnetic resonance imaging

## Abstract

**Purpose:**

To quantitatively assess the blood oxygen levels of the cerebral vein using quantitative susceptibility mapping (QSM), and to analyze the correlation between magnetic susceptibility value (MSV) and clinical laboratory indicators/cognitive scores in patients with Alzheimer’s disease (AD).

**Materials and Methods:**

Fifty-nine patients (21 males and 38 females) with clinically confirmed AD (AD group) and 22 control subjects (12 males, 10 females; CON group) were recruited. Clinical data and laboratory examination indexes were collected. All patients underwent Mini-mental State Examination, Montreal Cognitive Assessment, Clock Drawing Task, and Activity of Daily Living Scale test, as well as a routine MRI and enhanced gradient echo T2 star weighted angiography (ESWAN).

**Results:**

Higher cerebral venous MSV was observed in AD group compared to CON group, significant differences were observed for bilateral thalamus veins and left dentate nucleus veins. The MSV of bilateral thalamus veins, bilateral internal cerebral veins, and bilateral dentate nucleus veins had significant negative correlation with Mini-mental State Examination score; the MSV of bilateral thalamus veins, bilateral dentate nucleus veins, right septal vein had a significant negative correlation with Montreal Cognitive Assessment scores; a significant negative correlation between the MSV of bilateral thalamus veins, left dentate nucleus vein, right septal vein and the Clock Drawing Task score; the MSV of bilateral thalamus veins, left dentate nucleus vein had a significant negative correlation with Activity of Daily Living Scale score. The MSV of left dentate nucleus vein was positively correlated with the course of the disease, the MSV of bilateral septal vein were positively correlated with the total cholesterol, and the MSV of left septal vein had a positive correlation with LDL.

**Conclusion:**

Decreasing cerebral venous oxygen level in AD patients may affect cognitive status, and associated with the deterioration of the disease in AD patients.

## Introduction

Alzheimer’s disease (AD) is a progressive neurodegenerative disease and the most prevalent type of dementia in the elderly (Gong et al., 2019). AD is characterized by progressive cognitive and functional deficits and behavioral changes. Neuropathological features of AD include neuronal loss caused by neurofibrillary tangles (NFTs) and senile plaques formed by the deposition of β-amyloid protein (Aβ) in the brain (Harrington, 2012).

Previous studies have shown that cerebral blood flow is decreased in AD patients (Mak et al., 2014). Cerebral metabolic ratio of oxygen may decrease with the long-term cerebral blood perfusion insufficiency, which may affect the metabolism of neurons. Nevertheless, it remains unclear whether the decrease of cerebral oxygen content is associated with the cognitive decline in patient with AD. Different MRI sequences are currently applied to quantify the changes in cerebral oxygen and calculate oxygen extraction fraction (OEF) (An et al., 2001). Local OEF can be calculated based on the changes in venous phase values of susceptibility weighted imaging (SWI), which has just been applied to clinical work (Barnes and Haacke, 2009; Kesavadas et al., 2010; Zaitsu et al., 2011; Li et al., 2013; Messina et al., 2014).

Over the recent years, it has been found that SWI has some shortcomings in quantitative measurement. φ value is a parameter reflecting the magnetic susceptibility of tissues. Yet, due to the effect of “dipole field,” the accuracy and repeatability of measurement of φ value are not high enough (Liu et al., 2015). In order to improve the shortcomings related to quantitative measurement of SWI, a new sequence known as quantitative susceptibility mapping (QSM) was designed. It is a non-invasive magnetic resonance imaging technique that generates a linearly proportional, volumetric image based on the magnetic susceptibility of the subject tissue (Derry and Kent, 2017). This sequence measures the magnetic susceptibility of tissues rather than the φ value, which solves the problem of measuring the φ value in algorithm. The intensity of QSM signal depends on the amount and concentration of deoxyhemoglobin in tissues and the diameter of blood vessels (Mukherjee et al., 2003), QSM can reliably calculate the oxygen saturation of human cerebral veins (Fan et al., 2014) and obtain the magnetic susceptibility value (MSV), provide information on oxygen metabolism (Jensen-Kondering and Böhm, 2013). To our previous literature review, there was no studies reported were found on cerebral venous oxygen level for patients with AD.

Based on the above factors, the purpose of this study was to quantitatively evaluate the cerebral venous oxygen level in AD patients by using QSM magnetic susceptibility. We also further analyzed the correlation between cerebral venous oxygen level and cognitive status in order to understand the mechanisms underlying changes of cerebral oxygen level in AD patients, and hoping to identify the imaging indicators for monitoring the evaluation of AD patients and further clinical intervention therapy.

## Materials and Methods

### Subjects

Fifty-nine patients with clinically confirmed AD (AD group) were recruited in the Neurology Department between September 2015 and December 2018. Subjects were enrolled based on National Institute of Neurological and Communicative Disorders and Stroke and the Alzheimer’s disease and Related Disorders Association (NINCDS/ADRDA) criteria for probable AD (McKhann et al., 1984, 2011). The exclusion criteria: (a) other forms of dementia, such as frontotemporal dementia, dementia with Lewy bodies, vascular dementia and Parkinson’s disease dementia; (b) a history of stroke, brain tumor, serious head trauma, smoking, alcohol or drug dependence, and neurologic or psychiatric diseases; (c) large-vessel disease and diseases with white matter lesions. In addition, 22 elderly without cognitive impairment were recruited as control group (CON group). In this group of patients, the high signal intensity of white matter on MRI was below Fazekas-scale grade 2 or less than 2 static lacunar infarctions in the brain. Both AD group and CON group were right-handed.

Clinical data including gender, age, level of education were collected from all subjects. Course of the disease and laboratory examination indexes, including total cholesterol, triglycerides, high-density lipoprotein (HDL), low density lipoprotein (LDL), and homocysteine levels were collected from AD patients.

This retrospective study was approved by the hospital ethics committee. In addition, all patients signed the informed consent.

### MR Image Acquisition

Routine MRI and enhanced gradient echo T2 star weighted angiography (ESWAN) were performed using the GE signa HDXT 3.0T MRI system with 8-channel head coil. All the scan sequence parameters were set the same. The specific scanning sequence and parameters are shown in Table 1.

**TABLE 1:** MRI scan parameters.

**Sequence**	**TR (ms)**	**TE (ms)**	**Interval (mm)**	**FOV (cm × cm)**	**Matrix**	**Flip angle (°)**	**Bandwidth (kHz)**
T1WI	2500	25	1	22 × 19.8	320 × 256	/	31.25
T2WI	5000	118	1	22 × 19.8	320 × 256	/	31.25
ESWAN	36	3.6; 7.8; 11.9; 16.1; 20.3; 24.4; 28.6; 32.8	0	24 × 24	256 × 256	20	31.25

### Image Post-processing

The ESWAN raw data was saved and transmitted to a personal computer in DICOM format. Image post-processing was performed by using signal processing in nuclear magnetic resonance (SPIN) software provided by Wayne State University. Firstly, the original phase map and magnitude map of ESWAN were further processed by susceptibility weighted imaging mapping (SWIM) module in SPIN software to obtain QSM. The processing steps were as follows: (1) brain extraction: skull removal were carried out, and the artifacts on phase map were eliminated by compound threshold: BET = 0.2; (2) phase unwrapping; (3) phase quality map: kernel size = 6, threshold = 0.05; (4) background field removal: sophisticated harmonic artifact reduction for phase data (SHARP); (5) the standard inversion filtering was applied to Fourier transform of high-pass filter phase map.

### ROI Selection

**FIGURE 1:**
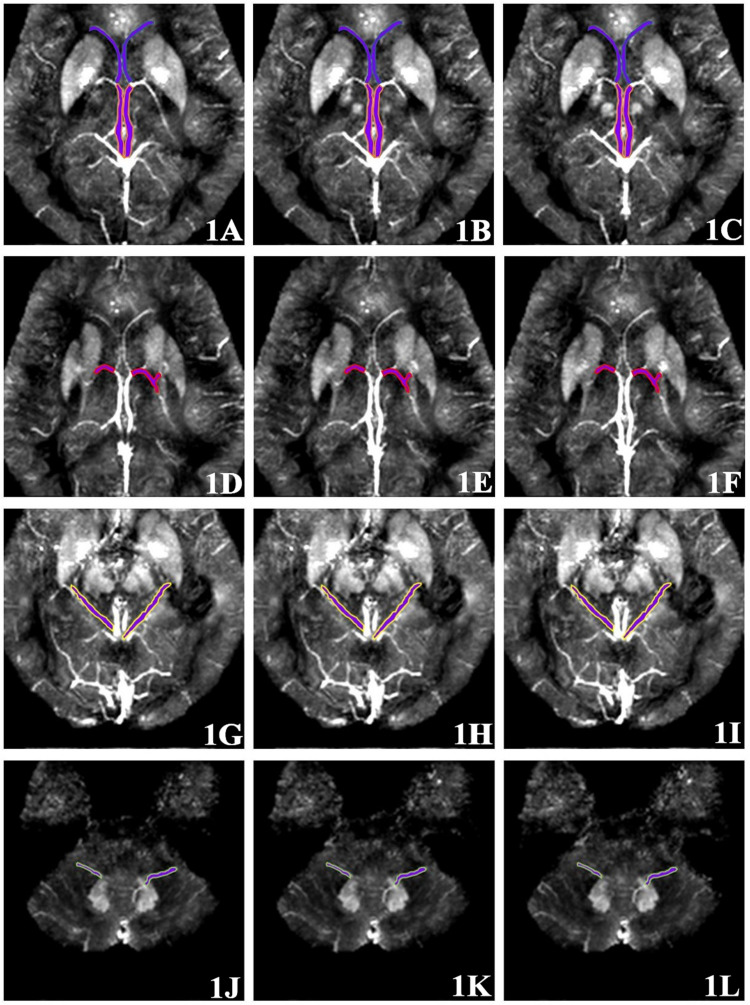
Region of interest (ROI) selection of cerebral vein. **(1A–1C)** Three continuous slices; the SV and ICV are shown front and back. **(1D–1F)** Three continuous slices showing the TV. **(1G–1I)** Three continuous slices showing the BV. **(1J–1L)** Three continuous slices showing the DNV.

Quantitative measurement of MSV of region of interest (ROI) in magnetic sensitive maps was analyzed using SPIN software. As shown in Figure 1, the ROI included bilateral internal cerebral veins (ICV), septal veins (SV), thalamus veins (TV), basal veins (BV) and dentate nucleus veins (DNV). Briefly, the ROIs were manually drawn along the boundary of the full length of the vein at the maximum slice that clearly displayed the vein. Three consecutive slices were then measured at the same location and averaged, avoiding the influence of air-cranial junction and ventricular system. Due to the small range of the SV and DNV in some patients, it could not be clearly displayed at three consecutive slices, then the same part was repeatedly measured three times and averaged. In order to ensure maneuverability of measurement, the ROI should select vessels with clear display and relatively fixed. The ROIs were manually drawn by two neuro-radiologists (neuro-radiologist 1 with 10 years of experience, neuro-radiologist 2 with 5 years of experience) in the same manner. To evaluate inter-observer reliability, the intra-class correlation coefficients (ICCs) were calculated, ICC > 0.75 was considered with inter-observer stability.

### Cognitive Scale

Neurological and cognitive functions in all subjects, including Mini-mental State Examination (MMSE), Montreal Cognitive Assessment (MoCA), Clock Drawing Task (CDT), and Activity of Daily Living Scale (ADL) were analyzed by a senior neurologist.

### Statistical Analysis

Data analyses were performed using statistical package for social science (SPSS) version 20.0 and R version 3.5.3. ICC test was calculated to evaluate inter-observer reliability. Independent-samples *t*-test (for normally distributed data) or Mann–Whitney *U* test (for non-normally distributed data) was used to compare the MSV of cerebral venous between AD group and CON group. Spearman correlation analysis was performed on the MSV of cerebral venous and the MMSE score, MoCA score, CDT score, and ADL score. The correlation between clinical and laboratory data of the AD group and MSV were performed by Pearson correlation analysis (for normally distributed data) or Spearman correlation analysis (for non-normally distributed data). False discovery rate (FDR) (Benjamini-Hochberg method) was further adopted to correct for multiple comparisons. *P* < 0.05 was considered statistically significant.

## Results

### Subject Characteristics

The clinical and cognitive data were summarized in Table 2. There was no significant difference in gender, age or years of education between the two groups (*p* > 0.05). The MMSE score, MoCA score, CDT score and ADL score in AD group were significantly different from that in CON group (*p* < 0.001).

**TABLE 2:** Clinical data.

**Characteristics**	**AD group (*n* = 59)**	**CON group (*n* = 22)**	***p*-value**
Gender (M/F)	21/38	12/10	0.123
Age^*a*^	71.12 ± 9.21	67.86 ± 8.60	0.154
Years of education^*b*^	9.00 (3.00)	9.00 (6.00)	0.534
Course of the disease^*a*^	3.68 ± 2.42	–	–
MMSE^*b*^	19.00 (9.00)	30.00 (0.00)	<0.001*
MoCA^*b*^	14.00 (8.00)	30.00 (0.25)	<0.001*
CDT^*b*^	2.00 (2.00)	4.00 (0.00)	<0.001*
ADL^*b*^	27.00 (11.00)	14.00 (0.00)	<0.001*
Total cholesterol^*b*^	5.29 (1.42)	–	–
Triglycerides^*b*^	1.02 (0.60)	–	–
HDL^*a*^	1.46 ± 0.36	–	–
LDL^*a*^	2.86 ± 0.87	–	–
Homocysteine^*b*^	14.23 (5.60)	–	–

^*a*^Data are expressed as mean value ± standard deviations. ^*b*^Data are expressed as median (inter-quartile range). *P < 0.05.

### Cerebral Vein MSV in AD Group and CON Group

**FIGURE 2:**
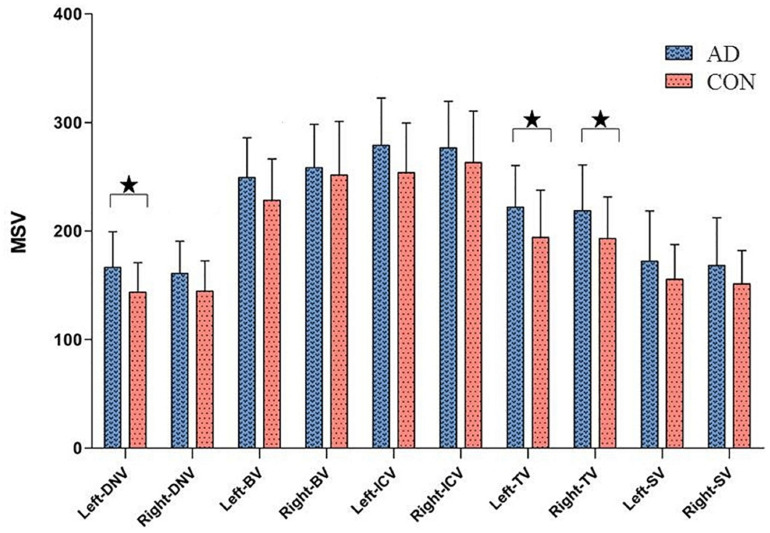
Comparisons of cerebral vein MSV between AD group and CON group, ^★^*P* < 0.05.

The ICC of the two neuro-radiologists were 0.809∼0.929. As shown in Figure 2 and Table 3, higher cerebral vein MSV were detected in AD group compared to CON group; significant differences were observed in bilateral TV (pL = 0.043, pR = 0.043), and left DNV (*p* = 0.028).

**TABLE 3:** Cerebral vein MSV between AD group and CON group.

**Region**	**AD group**	**CON group**	**Original *p*-value**	**FDR corrected *p*-value**
Left-DNV^*a*^	166.44 ± 32.88	143.65 ± 27.13	0.002	0.028*
Right-DNV^*a*^	161.12 ± 29.37	144.64 ± 27.91	0.025	0.059
Left-BV^*a*^	249.37 ± 36.54	228.23 ± 38.35	0.032	0.059
Right-BV^*b*^	260.00 (54.00)	232.00 (61.75)	0.199	0.249
Left-ICV^*b*^	283.00 (59.50)	243.50 (75.75)	0.036	0.059
Right-ICV^*a*^	276.58 ± 43.04	263.14 ± 47.30	0.252	0.252
Left-TV^*a*^	221.93 ± 38.57	194.14 ± 43.69	0.013	0.043*
Right-TV^*a*^	218.80 ± 42.11	193.10 ± 38.30	0.012	0.043*
Left-SV^*b*^	165.00 (55.00)	156.50 (20.75)	0.226	0.251
Right-SV^*b*^	156.00 (61.00)	147.00 (42.75)	0.130	0.186

^*a*^Data are expressed as mean value ± standard deviations. ^*b*^Data are expressed as median (inter-quartile range). *P < 0.05.

### Correlation Analysis Between the MSV of Bilateral Cerebral Veins and the Cognitive Scores in AD Group

**FIGURE 3:**
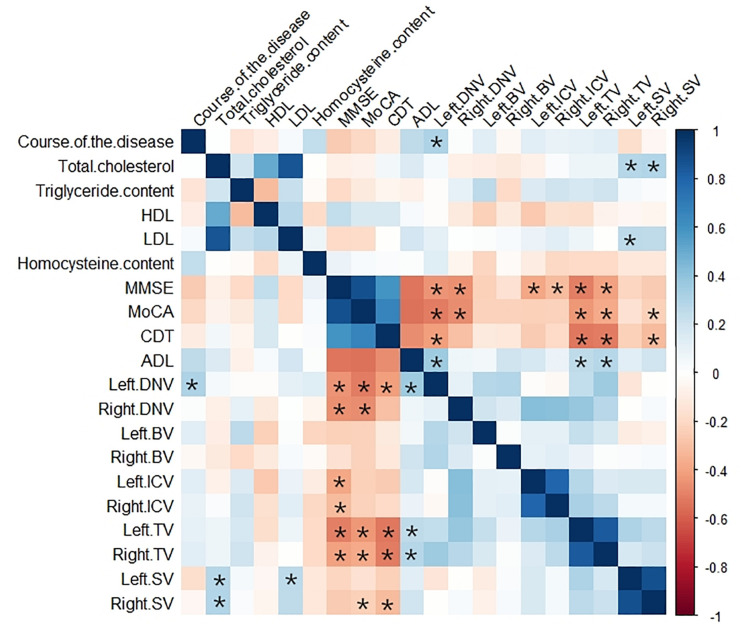
Heat map showing the correlation analysis between MSV of cerebral veins and clinical data, laboratory examination indexes, MMSE score, MoCA score, CDT score, ADL score in AD group. Color bar on the right side displays the value of the correlation coefficient (higher from –1 to 1, and from red to blue). *Significant correlation.

**FIGURE 4:**
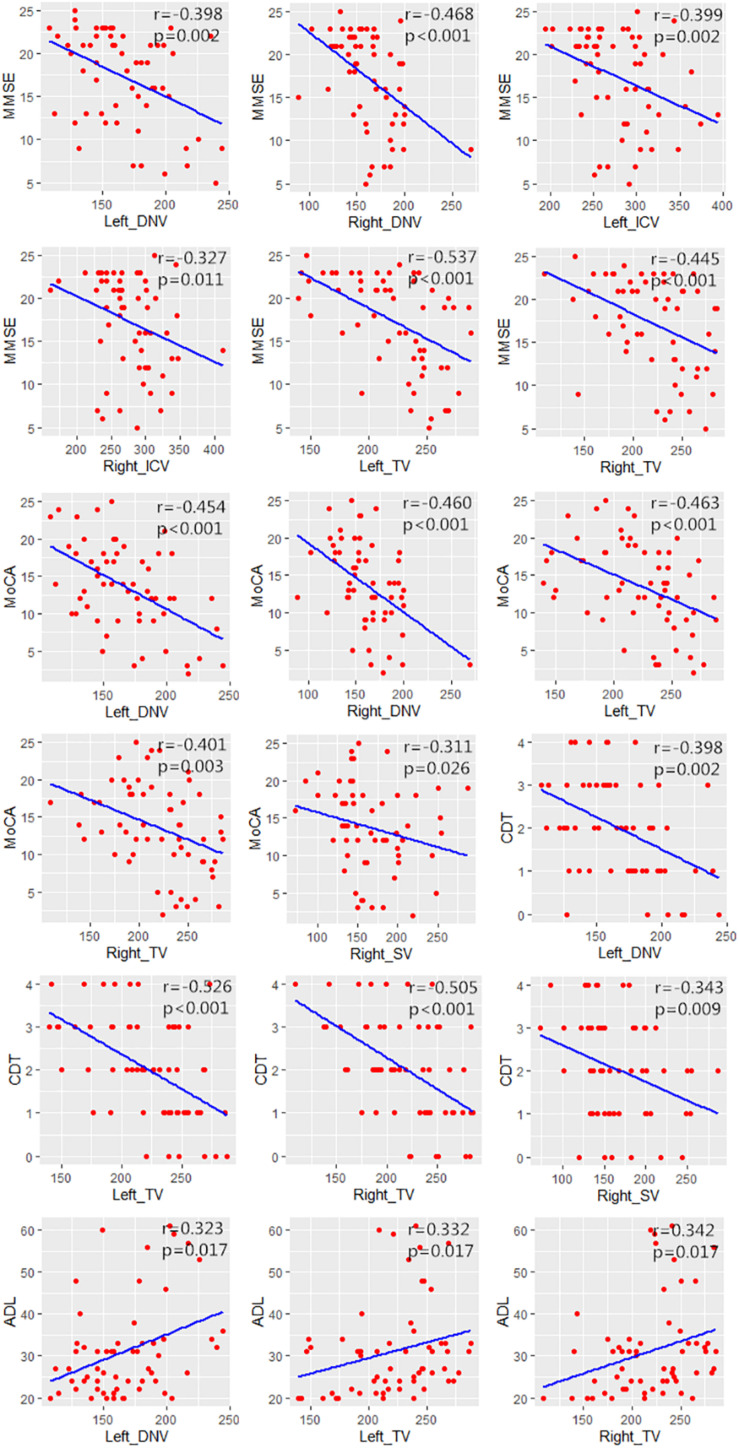
Scatter plots showing the significant correlation between MSV of cerebral veins and MMSE score, MoCA score, CDT score, ADL score.

The correlation analysis results between MSV of cerebral veins and MMSE score, MoCA score, CDT score, and ADL score were represented by heat maps (Figure 3), and the significant correlation were represented by scatter plots (Figure 4). The MSV of bilateral TV (rL = −0.537, *p* < 0.001; rR = −0.445, *p* < 0.001), bilateral ICV (rL = −0.399, *p* = 0.002; rR = −0.327, *p* = 0.011) and bilateral DNV (rL = −0.398, *p* = 0.002; rR = −0.468, *p* < 0.001) were all negatively correlated with the MMSE scores. Among these, the MSV of left TV showed moderate correlation, while others showed low correlation with MMSE. Furthermore, the MSV of bilateral TV (rL = −0.463, *p* < 0.001; rR = −0.401, *p* = 0.003), bilateral DNV (rL = −0.454, *p* < 0.001; rR = −0.460, *p* < 0.001) and right SV (*r* = −0.311, *p* = 0.026) were negatively correlated with the MoCA scores. In addition, there was a significant negative correlation between the MSV of bilateral TV (rL = −0.526, *p* < 0.001; rR = −0.505, *p* < 0.001), left DNV (*r* = −0.398, *p* = 0.002), the right SV (*r* = −0.343, *p* = 0.009) and the CDT score; moderate correlation was observed between bilateral TV and CDT score, while the MSV of left DNV and right SV displayed low correlation with CDT score. Additionally, the MSV of bilateral TV (rL = 0.332, *p* = 0.017; rR = 0.342, *p* = 0.017), and the left DNV (*r* = 0.323, *p* = 0.017) were positively correlated with ADL score.

### Correlation Analysis Between MSV of Cerebral Veins and Clinical and Laboratory Data in AD Group

**FIGURE 5:**
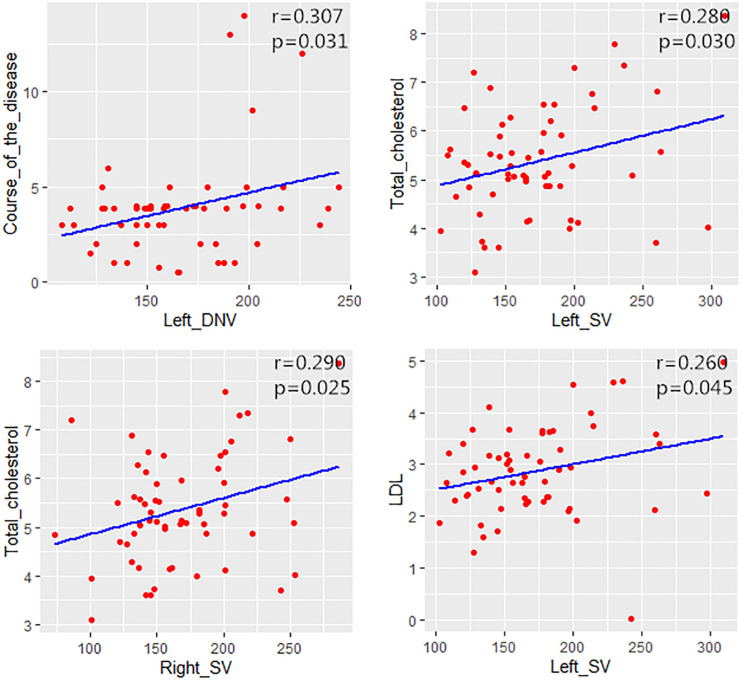
Scatter plots showing the correlation between MSV of cerebral veins and the course of the disease, total cholesterol and LDL in AD group.

The correlation analysis results between MSV of cerebral veins and clinical and laboratory data in AD group were represented by heat map (Figure 3). The MSV of left DNV (*r* = 0.307, *p* = 0.031) had a positive correlation with the course of the disease. The MSV of bilateral SV (rL = 0.280, *p* = 0.030; rR = 0.290, *p* = 0.025) were positively correlated with the total cholesterol, and the MSV of left SV (*r* = 0.260, *p* = 0.045) had a positive correlation with LDL (Figure 5). However, no correlation was found between MSV of cerebral veins and HDL, homocysteine levels (*p* > 0.05).

## Discussion

Venous oxygen level is an important factor for evaluating brain development and function. Under normal conditions, the brain consumes 20% of the total energy of the body through oxidative metabolism (Gallagher et al., 1998). The measurement of venous oxygen is crucial for analyzing the neuronal activity and brain physiology during aging and regulation of brain disease status (Donahue et al., 2009). Under normal conditions, blood oxygen level is in a relatively constant state. Nevertheless, abnormal condition, such as cerebrovascular and neurodegenerative diseases or brain trauma can affect brain oxygen levels, which may further lead to a decline of cognitive level. Previous studies have shown that vascular risk factors can participate in the pathogenesis and progression of AD (Li et al., 2011; Zhu et al., 2014; Inzelberg et al., 2015).

Previous studies have found that AD is associated with blood–brain barrier (BBB) dysfunction. BBB is an important structure that maintains the microenvironment homeostasis of the central nervous system. It can prevent pathogens and other macromolecules from entering the brain through blood circulation, excrete metabolites from brain tissues, and participate in the cerebrospinal fluid regulation and immune response (Halliday et al., 2016). BBB dysfunction leads to the accumulation of metabolites in blood vessels, increases oxygen consumption, and further reduces the venous oxygen levels. Structural alterations, atherosclerotic lesions, and impaired hemodynamic may occur in the cerebral vessels of AD patients (Meyer et al., 2008), and the peripheral nerve tissue is accompanied by axonal loss and myelin dissolution (Gouw et al., 2008), which put the brain tissue of AD patients in a hypoxic state.

In this study, QSM was first used to quantify blood oxygen levels in cerebral veins of AD patients. We found that the oxygen level of bilateral cerebral veins in AD group was generally lower than that in CON group, in addition, the proportion of deoxyhemoglobin increased, which is consistent with previous studies. However, significant differences were observed only for left DNV and bilateral TV, thus suggesting that besides the relationship between MSV and the content of deoxyhemoglobin in blood vessel, diameter of blood vessel is another important factor for the diagnosis of AD patients. Blood vessels with the small diameter are easily affected by the slice and partial volume effect during the measurement process, which leads to some deviations in the measurement results.

Furthermore, our data indicated that the MSV of bilateral TV, bilateral ICV and bilateral DNV were all negatively correlated with MMSE scores, and there was negative correlation between the MSV of bilateral TV, bilateral DNV, right SV and the MoCA scores. In addition, the MSV of bilateral TV, left DNV and right SV were all negatively correlated with CDT scores; while the MSV of bilateral TV, left DNV were positively correlated with ADL scores. Thus, to sum up, higher MSV indicates lower venous oxygen level, the lower the cognitive scores, and the worse the cognitive level. Because AD patients have low venous oxygen level and AD is a chronic progressive disease, the brain tissue may be in a state of long-term hypoxia, which will inevitably lead to the decline of brain function and consequently reduce the cognitive level.

Our findings showed the MSV of the left DNV was positively correlated with the course of disease, indicating that the longer course of disease led to lower oxygen levels of the left DNV. The development of AD is an irreversible pathological process. Consequently, the longer course of AD, leads to more serious conditions, while the decrease of cerebral venous oxygen level is positively correlated with the deterioration of the disease.

In this study, the MSV of bilateral SV were positively correlated with the total cholesterol, and the MSV of left SV had a positive correlation with LDL. We suggested that dyslipidemia influences the cerebral blood perfusion through vascular disease, further leads to decreasing of cerebral oxygen level. Previous studies have reported that, dyslipidemia was a key factor of metabolic syndrome, and increases the risk of AD (Gentile et al., 2020). Wingo et al. (2019) found that elevated LDL levels were associated with higher probability of having early-onset AD, this novel findings highlight the important role of LDL in AD pathogenesis. Besides, increased plasma homocysteine level is a strong, independent risk factor for the development of AD (Seshadri et al., 2002). However, no correlation was found between MSV of cerebral veins and homocysteine levels.

The present study has some limitations: (1) this study was a retrospective study and our subjects were all inpatients, who consulted our hospital due to obvious symptoms. The course of the disease was relatively long and the symptoms were relatively severe. Also, there were few patients with mild cognitive impairment who were not included in this study, which should be addressed by further research. (2) The AD group and CON group were of different size, thus, larger sample size is needed to reduce errors. (3) The ROI in this study was manually placed, which will inevitably lead to certain errors.

## Conclusion

Decreasing cerebral venous oxygen level in AD patients may affect cognitive status, and associated with the deterioration of the disease in AD patients.

## Data Availability Statement

The original contributions presented in the study are included in the article/Supplementary Material, further inquiries can be directed to the corresponding author/s.

## Ethics Statement

The studies involving human participants were reviewed and approved by the Ethics Committee of First Affiliated Hospital of Dalian Medical University. The patients/participants provided their written informed consent to participate in this study. Written informed consent was obtained from the individual(s) for the publication of any potentially identifiable images or data included in this article.

## Author Contributions

YL, JD, and YM conceived the study and participated in its design, data collection, and drafting of the manuscript. WW, BG, and ST participated in statistical analysis and software. QS and NZ participated in image reconstruction and data analysis. CD, ZL, and LX participated in the manuscript revise. The authors read and approved the final manuscript.

## Conflict of Interest

LX was employed by GE Healthcare. The remaining authors declare that the research was conducted in the absence of any commercial or financial relationships that could be construed as a potential conflict of interest.

## Abbreviations

AD: Alzheimer’s disease
NFTs: neurofibrillary tangles
A β: β-amyloid protein
OEF: oxygen extraction fraction
SWI: susceptibility weighted imaging
QSM: quantitative susceptibility mapping
HDL: high-density lipoprotein
LDL: low-density lipoprotein
ESWAN: enhanced gradient echo T2 star weighted angiography
SPIN: signal processing in nuclear magnetic resonance
SWIM: susceptibility weighted imaging mapping
SHARP: sophisticated harmonic artifact reduction for phase data
MSV: magnetic susceptibility value
ROI: region of interest
ICV: internal cerebral vein
SV: septal vein
TV: thalamus vein
BV: basal vein
DNV: dentate nucleus vein
MMSE: Mini-mental State Examination
MoCA: Montreal Cognitive Assessment
CDT: Clock Drawing Task
ADL: Activity of Daily Living Scale
FDR: false discovery rate
BBB: blood–brain barrier.
