# Supplementary material for: Correlation Between Cerebral Venous Oxygen Level and Cognitive Status in Patients With Alzheimer’s Disease Using Quantitative Susceptibility Mapping
Source: Front Neurosci. 2021 Jan 18;14:570848. doi: 10.3389/fnins.2020.570848 (PMC7848136; doi:10.3389/fnins.2020.570848)
Supplement: Supplementary file 1 [file Data_Sheet_1.PDF]

NO.	Group	L_BV	R_BV	L_ICV	R_ICV	L_TV	R_TV	L_SV	R_SV	L_DNV	R_DNV
sub001	1	279	288	255	263	140	138	131	131	165	185
sub002	1	274	247	223	243	239	262	190	222	204	102
sub003	1	259	333	236	243	172	159	135	145	152	153
sub004	1	213	252	298	301	174	154	154	135	140	127
sub005	1	198	298	243	236	213	213	261	250	166	167
sub006	1	249	234	256	236	142	109	139	131	158	160
sub007	1	185	169	195	213	161	179	123	145	108	155
sub008	1	202	312	290	292	192	232	191	200	150	153
sub009	1	164	266	259	244	169	172	127	85	158	134
sub010	1	211	235	234	253	206	250	103	101	198	133
sub011	1	229	259	295	244	213	213	180	187	114	171
sub012	1	324	250	314	293	248	193	141	122	160	153
sub013	1	255	235	254	284	217	194	165	137	178	181
sub014	1	287	292	343	343	227	191	152	156	128	195
sub015	1	219	166	235	287	185	221	148	142	145	122
sub016	1	312	232	313	307	227	208	154	182	191	170
sub017	1	233	298	364	345	205	161	298	254	169	147
sub018	1	280	243	305	266	273	245	197	180	180	194
sub019	1	210	282	309	314	269	236	203	201	125	168
sub020	1	285	246	257	263	239	232	124	73	145	147
sub021	1	245	262	272	266	214	199	146	141	134	148
sub022	1	292	164	394	337	239	243	199	200	137	200
sub023	1	237	221	251	265	207	184	114	127	159	123
sub024	1	238	285	348	338	278	282	152	150	216	199
sub025	1	278	232	253	261	255	266	168	136	188	167
sub026	1	270	296	244	253	243	241	200	212	150	143
sub027	1	222	199	326	346	246	242	176	137	112	190
sub028	1	245	260	298	303	247	233	181	168	185	149
sub029	1	230	240	242	244	265	284	243	253	145	148
sub030	1	218	187	230	228	219	189	310	287	122	126
sub031	1	258	293	201	163	193	204	128	101	193	128
sub032	1	286	268	314	330	232	204	182	133	173	194
sub033	1	268	268	229	247	191	189	198	161	145	168
sub034	1	278	289	246	268	286	286	146	150	176	198
sub035	1	223	243	242	261	255	252	178	143	158	154
sub036	1	254	287	303	299	193	197	139	151	156	145
sub037	1	260	290	292	287	252	275	110	133	239	159
sub038	1	245	274	299	321	266	258	165	156	180	185
sub039	1	265	197	289	304	263	266	230	201	161	159
sub040	1	298	315	288	291	288	278	166	159	189	179
sub041	1	224	219	284	266	218	208	145	142	129	154
sub042	1	236	247	298	299	177	175	167	201	197	119
sub043	1	307	220	244	174	148	197	179	185	235	142
sub044	1	263	265	304	307	239	249	120	182	244	269
sub045	1	264	298	266	262	236	238	178	168	174	166
sub046	1	238	272	317	243	194	143	163	172	131	187
sub047	1	153	312	251	237	254	233	263	248	199	163
sub048	1	310	231	312	325	246	265	186	201	178	160
sub049	1	222	255	374	290	248	251	260	243	128	187
sub050	1	274	299	283	296	235	243	120	155	226	185

sub051	1	262	353	357	411	244	284	215	198	184	200
sub052	1	252	280	257	230	269	224	237	218	217	178
sub053	1	186	257	331	289	221	223	108	120	205	141
sub054	1	235	240	236	267	209	219	133	148	149	146
sub055	1	301	269	266	234	241	241	213	206	202	89
sub056	1	265	208	286	299	268	276	183	196	152	199
sub057	1	216	301	301	312	147	140	129	144	128	132
sub058	1	287	278	203	232	207	180	155	149	156	168
sub059	1	240	231	256	263	150	186	156	167	134	142
sub060	0	220	259	271	272	255	233	144	145	141	151
sub061	0	181	195	208	216	173	172	148	144	134	163
sub062	0	216	223	247	222	145	151	126	122	95	146
sub063	0	257	329	293	316	229	193	155	175	154	144
sub064	0	194	208	224	282	211	169	135	147	112	81
sub065	0	209	223	238	263	188	174	156	124	123	88
sub066	0	204	199	320	291	224	239	184	167	94	135
sub067	0	174	213	214	208	131	142	83	99	165	154
sub068	0	225	357	310	343	262	251	160	183	133	176
sub069	0	288	298	183	186	143	152	205	210	123	121
sub070	0	259	235	218	242	151	209	147	149	121	132
sub071	0	297	257	286	281	206	202	165	123	160	121
sub072	0	212	232	250	231	165	197	130	134	165	133
sub073	0	243	318	322	321	218	203	157	179	133	191
sub074	0	187	218	233	290	226	205	145	148	123	151
sub075	0	212	224	240	253	198	231	159	139	145	148
sub076	0	213	200	311	299	234	240	175	169	199	195
sub077	0	179	222	210	212	134	134	110	100	173	154
sub078	0	223	346	313	341	267	259	165	178	167	177
sub079	0	279	288	179	189	141	153	245	214	177	149
sub080	0	249	232	221	232	154	142	165	151	161	143
sub081	0	300	259	295	299	216	197	163	131	162	129
